# Editorial: Rheological properties of functional and future foods

**DOI:** 10.3389/fnut.2026.1825401

**Published:** 2026-04-01

**Authors:** Gamze Yazar, Ilkem Demirkesen, Ozlem C. Duvarci, Cristina M. Rosell

**Affiliations:** 1 Department of Animal, Veterinary and Food Sciences, University of Idaho, Moscow, ID, United States; 2 Department of Animal Health, Food and Feed Research, General Directorate of Agricultural Research and Policies, Ministry of Agriculture and Forestry, Ankara, Türkiye; 3 Department of Chemical Engineering, Izmir Institute of Technology, Izmir, Türkiye; 4 Food and Human Nutritional Sciences Department, University of Manitoba, Winnipeg, MB, Canada; 5 Institute of Agrochemistry and Food Technology-Spanish National Research Council (IATA-CSIC), Paterna, Spain

**Keywords:** functional foods, quality optimization, rheology, sustainable foods, texture

The food industry has been focusing on developing sustainable and healthy foods to meet the growing food demand due to constantly increasing global population without undermining natural resources (1–4). A successful development of such products depends not only on their nutritional profile but also on their structural, textural, and sensory properties, which strongly influence consumer acceptance (5, 6). Rheology plays a central role in the design and performance of functional and future foods, as it governs not only processability and structural stability, but also texture perception, palatability, and ultimately physiological behavior during oral processing and digestion (6–8). During processing operations such as mixing, heating, cooling, homogenization, extrusion, drying, fermentation, enzymatic treatment, 3D printing, and high-pressure processing, food matrices experience molecular and conformational changes leading to microstructural and mechanical transformations (7, 9, 10). Such structural modifications affect viscosity, elasticity, yield stress, shear-thinning/-thickening, strain-stiffening/-softening, gelation and gel strength, and stability, all of which are key parameters in the design and optimization of food products (7, 8, 11, 12). By defining how food matrices flow, deform, and recover when exposed to external stresses, rheological characterization provides important information for food scientists in ingredient selection strategies to design, improve and optimize product quality, to select and optimize their manufacturing processes (7). In this context, rheology provides a fundamental bridge between ingredient composition, processing technologies, product performance, and consumer perception. Research on the rheological properties of functional and future foods offers valuable scientific insight for the development of innovative, nutritious, and sustainable food products from a broad range of raw materials and processing approaches. Therefore, this Research Topic highlights how the deliberate modulation of viscoelasticity and flow behavior enables the development of next-generation food products.

Hydrocolloids are used as structure-forming and stabilizing agents in various processing applications due to their ability to regulate rheology and texture (13). Omedi et al. investigated the effect of hydrocolloids on the rheological properties of pitaya fruit-based inks for 3D printing. 3D printing is among the techniques used to fabricate plant-based meat analogs (14, 15). The viscosity of the plant-based ink affects the printing process and the quality of the resulting matrix (16). Therefore, the study of Omedi et al. shows the possibility to manipulate the rheological properties of plant-based inks through the use of hydrocolloids.

Hydrocolloids are also used to modify the viscoelastic properties of starch gels (17, 18). Montes et al. studied the linear viscoelastic properties of corn starch-Hydroxypropyl Methylcellulose (HPMC) gels in comparison with wheat starch gel. Adding HPMC in corn starch at low percentages ranging from 1% to 1.5% resulted in gels that mimic the viscoelastic properties of wheat starch gel. The findings of this study are useful for designing gluten-free baked products (Montes et al.), while they can be also benefited in developing starch-based edible inks in the field of 3D food printing (19, 20).

Plant-based gels find a wide range of applications not only due to their tunable rheological properties, but also due their health benefits, low toxicity, biocompatibility, and biodegradability (21, 22). Trigueros et al. studied the rheological properties of the gels made from defatted canary seed flour. The supercritical carbon dioxide extraction was used as a sustainable alternative to the conventional hexane extraction in this study to simultaneously valorize canary seed oil and the resulting defatted flour. The higher elasticity found for the gel defatted with supercritical carbon dioxide revealed the potential of defatted canary seed flour as thickening or structuring agents (Trigueros et al.). Such structural functionality is largely linked to protein networks formed during processing, which become densely crosslinked and entangled in the absence of fat (23, 24).

Other studies in this Research Topic focused on improving the textural properties of gluten-free baked products through the use of dairy proteins. Shan et al. incorporated whey protein fibrils (prepared at pH 3.5 and pH 7) into the blend of rice and potato starches and compared the linear and non-linear viscoelastic properties of these gluten-free doughs to those of gluten doughs. The composite starch dough with added whey protein fibril at pH 7 showed similar η^*^(ω) and strain hardening properties to those of gluten, which was attributed to the formation of protein aggregates at pH 7. Graça et al. evaluated the impact of added yogurt and curd cheese (10% and 20%, w/w) on the rheological properties of gluten-free doughs. The strong linear correlations obtained between the rheological response of the dough and the quality traits of the resulting bread suggested improved loaf volume, firmness, and shelf-life in gluten-free breads with increasing levels of added dairy products. Added dairy proteins also enhance the nutritional quality of gluten-free products, that are mostly deficient in certain nutrients like dietary fiber, vitamins, and minerals (25).

The integration of rheological science into the formulation and processing of food systems offers transformative potential for the food industry. The studies presented in this editorial highlight the importance of rheology in plant-based food system development, including meat and dairy alternatives, as well as gluten-free baked products. The use of rheological principles by researchers and food manufacturers enables optimizing texture, structure, and overall quality in the end-products, assuring that they meet consumer expectations while promoting sustainability.
